# Supplementary material for: The interactome of intact mitochondria by cross-linking mass spectrometry provides evidence for coexisting respiratory supercomplexes
Source: Mol Cell Proteomics. 2017 Dec 8;17(2):216–32. doi: 10.1074/mcp.RA117.000470 (PMC5795388; doi:10.1074/mcp.RA117.000470)
Supplement: Supplemental Data [file supp_RA117.000470_133922_0_supp_23978_fzffwf.zip › spectra_annotation/mito_spectra_annotation/138-1-4-1-20-1.pdf]

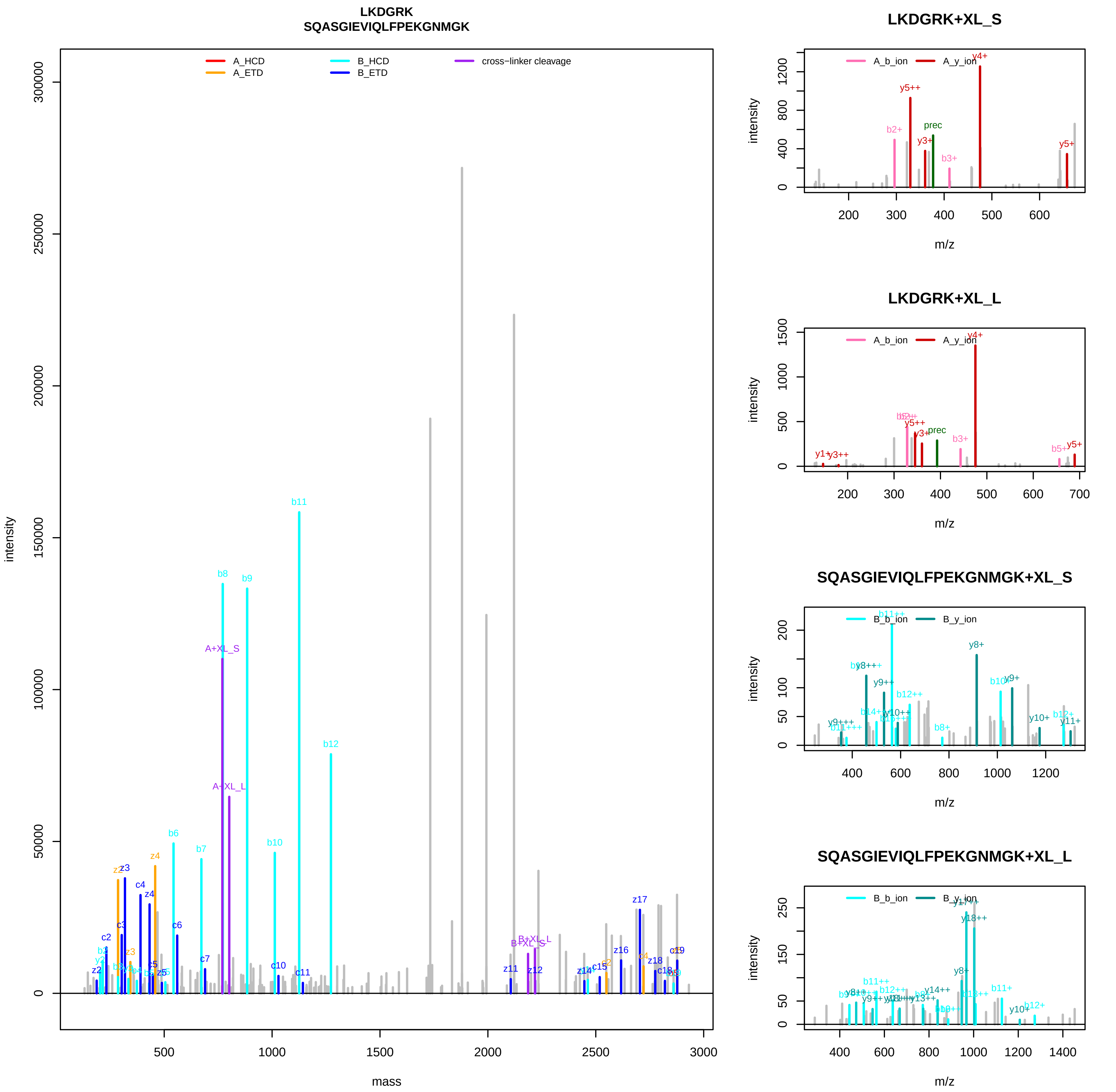

LKDGRK
SQASGIEVIQLFPEKGNMGK
A_HCD
A_ETD
B_HCD
B_ETD
cross−linker cleavage
intensity
mass
0
50000
100000
150000
200000
250000
300000
500
1000
1500
2000
2500
3000
b6
b7
b8
b9
b10
b11
b12
A+XL_S
A+XL_L
z4
z17
c19
LKDGRK+XL_S
A_b_ion
A_y_ion
y4+
y5++
b2+
y3+
prec
b3+
y5+
LKDGRK+XL_L
SQASGIEVIQLFPEKGNMGK+XL_S
B_b_ion
B_y_ion
y8+
SQASGIEVIQLFPEKGNMGK+XL_L
m/z
